# Foreskin trapped by zipper: a case report

**DOI:** 10.4076/1757-1626-2-6345

**Published:** 2009-07-27

**Authors:** Miguel Angel Arrabal-Polo, Salvador A Arias-Santiago, Maria Sierra Giron-Prieto, Miguel Arrabal-Martin

**Affiliations:** 1 Department of Urology, San Cecilio University Hospital (Avenida Doctor Oloriz 18)Granada (18012)Spain; 2 Department of Dermatology, San Cecilio University Hospital (Avenida Doctor Oloriz 18)Granada (18012)Spain; 3 Department of General Medicine, Virgen de las Nieves University Hospital (Avenida Fuerzas Armadas)Granada (18012)Spain

## Abstract

We present an 84 year-old-male patient with foreskin trapped by his zipper. After several failed attempts with scissors, screwdriver and others we practise an elliptic incision to resolve the problem. Foreskin injuries are frequent in children but are rare in adult men. There are some techniques described for solving the problem using scissors, screwdriver, traction and surgery.

## Introduction

Foreskin injuries are very rare in adult men, although it is frequent in children. There are some techniques to solve foreskin trapped by zipper.

## Case presentation

**Figure 1 & Figure 2.**
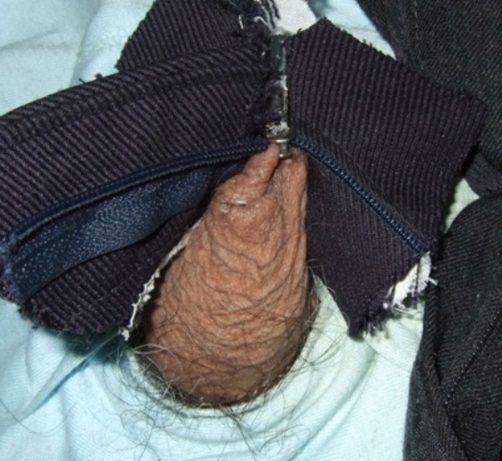

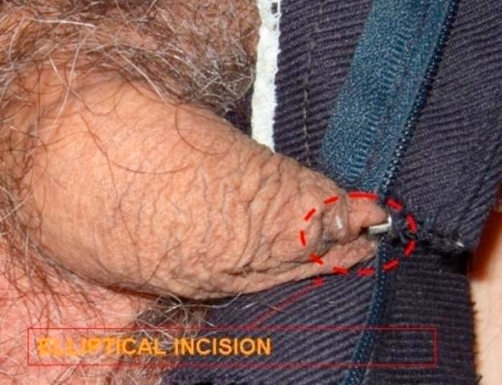
We can observe foreskin trapped by zipper.

An 84-year-old-Caucasian and Spanish-man presented at emergency service with a three hours history of a penile injury as his foreskin had been trapped by his zipper’s pants (Figure 1 & Figure 2). After several attempts, the patient could not release the foreskin of the zipper; he desisted and required an emergency evaluation by urologist as he had an intense pain with a mild haematoma and swelling. After several failed attempts using scissors, screwdriver and traction we decided to practise an elliptic incision (Elliptical incision on the skin of the penis and after continuous suture with absorbable thread) to resolve the emergency situation. Three weeks later a restitution ad integrum was observed, without urinary difficulty.

## Discussion

Foreskin injuries by zipper are relatively frequent in no circumcised children, however to the best of our knowledge, no cases in the elderly patient have been published in the literature, probably because patients achieves to release the penis of the zipper. The mean problem of this accident is the ischemia and necrosis of part of foreskin. Raveenthiran published a technique with a screwdriver to remove the zipper which has been used in 12 children successfully [1]. Mishra described a technique for releasing the foreskin of the zipper using ordinary wire cutter [2]. Others authors perform traction of the zipper [3]. It is usually ineffective the application of oil to sliding the zipper [4]. Finally elliptical incision has been described as definitive method [5]. All techniques described are suitable for solving the problem.
